# Spontaneous esophageal mucosal dissection in a patient with upper digestive bleeding and esophageal varices

**Published:** 2011-05-25

**Authors:** L Negreanu, LC Tribus, M Purcarea, C Fierbinteanu Braticevici

**Affiliations:** ‘Carol Davila’ University of Medicine and Pharmacy, Bucharest; Internal Medicine and Gastroenterology Department, Emergency University Hospital BucharestRomania

**Keywords:** spontaneous esophageal dissection, portal hypertension, endoscopy

## Abstract

We present a case of mucosal esophageal dissection in a 44–year–old patient with alcoholic cirrhosis admitted for upper digestive bleeding. The endoscopic aspect was of chronic mucosal dissection in the esophagus and 3rd degree esophageal varices with red signs.

To our knowledge, it is the only case with spontaneous esophageal mucosal dissection and portal hypertension with esophageal varices.

## Case presentation

A 44–year–old woman with alcoholic cirrhosis was admitted in our department for hematemesis. The diagnosis of alcoholic Child B cirrhosis was made several years before in another hospital. At that moment, an upper endoscopy was performed and grade two esophageal varices and moderate portal hypertension gastritis were mentioned, but no other findings. The patient took intermittent treatment with diuretics and beta–blockers but she continued drinking.

At admission, the patient was pale, hemodynamically unstable with orthostatic hypotension Complementary tests included a blood test (leukocytosis with neutrophilia and severe anemia).   

Aggressive fluid therapy, blood transfusion and octreotide infusion were started and an endoscopy was performed during the first 12 hours from admission. 

Upper endoscopy found a large mucosal longitudinal dissection with a double lumen apparent in the lower half of the esophagus. Large grade 3 varices with red signs were found. One of the variceal cords was particularly impressive being ‘suspended’ in a mucosal fold completely separated from the submucosa.

**Figure 1:**
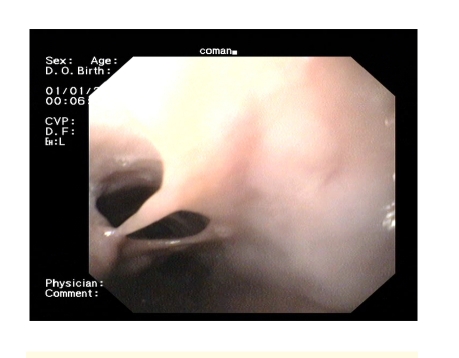
Proximal aspect–dissection

**Figure 2:**
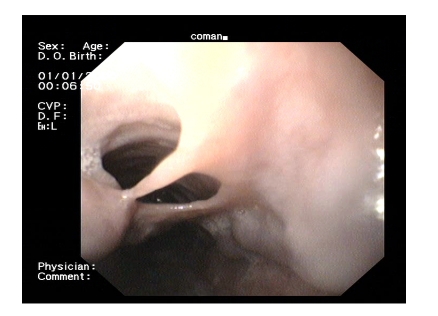
Proximal aspect–Double lumen

**Figure 3:**
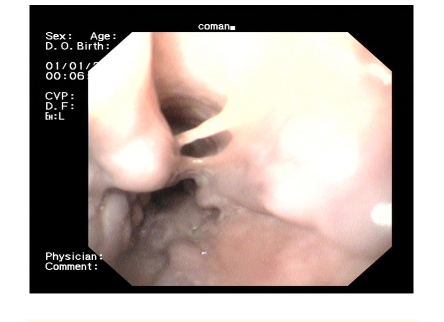
Variceal cord in the crease dissection

**Figure 4:**
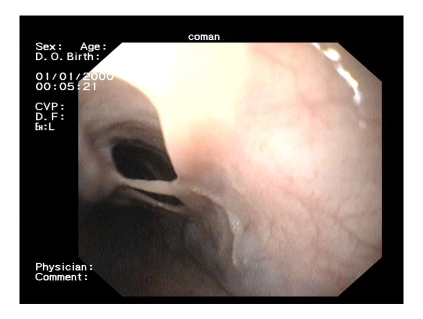
Double lumen

A contrast swallow study was done to obtain more information about the possibility of an esophageal perforation; it has demonstrated an extra luminal pool of contrast in a well–defined tubular false lumen with no evidence of contrast leak in the mediastinum but no clear aspect of a ‘double barrel’ esophagus.

Without an esophageal perforation demonstrated, we continued the conservative management (octreotide infusion for 48 hours, PPI, blood transfusions, broad–spectrum antibiotics and parenteral nutrition).

We discussed the utility of band ligation of the esophageal varices, a procedure that carried some risks especially for the variceal cord in the dissection esophageal fold, but the patient refused the procedure. Another endoscopic procedure consisting in the incision of the septum of dissection was not an option in our case or the placement of expandable esophageal stent. 

### Discussion

Spontaneous intramural dissection of the esophagus (SIDE) is a rare finding. Its etiology remains speculative.  SIDE can appear in several conditions like eosinophilic esophagitis, chronic anticoagulation, and secondary to endoscopic maneuvers especially after sclerotherapy [1,2,3,4]. In addition, it can be idiopathic.Probably the dissection results from an intramural hematoma in most cases.  

The most common presenting symptoms are sudden retrosternal pain, upper gi bleeding with hematemesis, odynophagia, dysphagia, and back pain [3].

Upper gastrointestinal endoscopy is a useful diagnostic test when radiological examinations (hydrosoluble contrast esophagogram, computed tomography, or magnetic resonance imaging) have excluded perforation. Intramural parietal dissection characteristically appears on barium swallow as a ‘double–barrelled’ esophagus related to a thin radiolucent mucosal membrane separating the false and true lumens [1,2,3].

It is a benign disease that, despite its alarming endoscopic appearance, responds well to conservative treatment and has an excellent pronostic. In some situations emergency surgical treatment is required (esophageal perforation with mediastinitis, massive bleeding, and abscesses). In some instances endoscopic treatment with the incision of the dissection fold or the placement of an esophageal prosthesis is an option [5]. 

Our patient has never had endoscopic therapy before. She had a good evolution after conservative treatment and was discharged home. 
